# Supplementary material for: The Prostate Cancer Therapy Enzalutamide Compared with Abiraterone Acetate/Prednisone Impacts Motivation for Exploration, Spatial Learning and Alters Dopaminergic Transmission in Aged Castrated Mice
Source: Cancers (Basel). 2021 Jul 14;13(14):3518. doi: 10.3390/cancers13143518 (PMC8304001; doi:10.3390/cancers13143518)
Supplement: Supplementary file 1 [file cancers-13-03518-s001.zip › cancers-1189828-supplementary.pdf]

cancers

MDPI

Supplementary Materials: The Prostate Cancer Therapy Enzalutamide Compared with Abiraterone Acetate/Prednisone Impacts Motivation for Exploration, Spatial Learning and Alters Dopaminergic Transmission in Aged Castrated Mice

Celeste Nicola, Martine Dubois, Cynthia Campart, Tareq Al Sagheer, Laurence Desrues, Damien Schapman, Ludovic Galas, Marie Lange, Florence Joly and Hélène Castel

Figures, Tables and Schemes

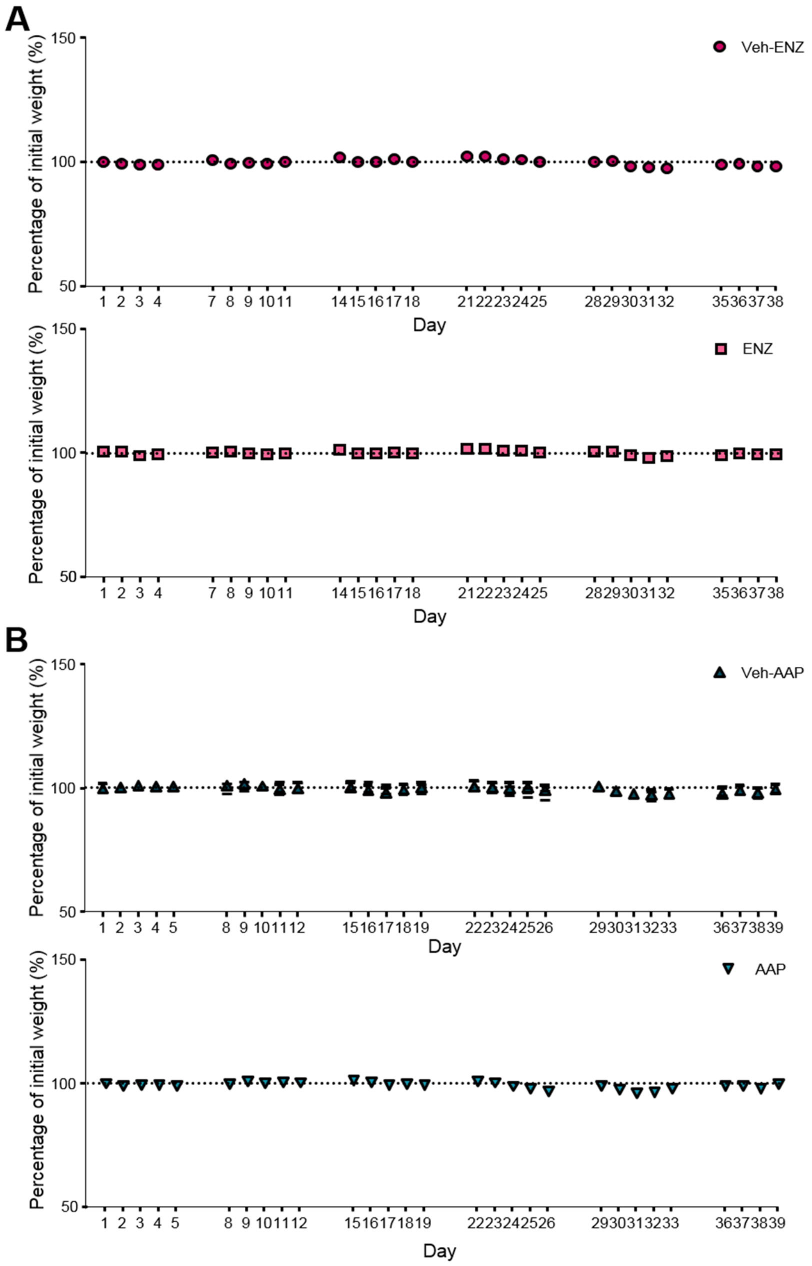

Figure S1. ENZ or AAP have no impact on body weight gain. Note. (**A**) Body weight gain curves of ENZ-treated mice compared with Veh-ENZ. Note. Statistical analysis was performed between

vehicle (n = 14) and treated (n = 16) data using one-way ANOVA with repeated measures, followed by Sidak's multiple comparison test. Data are expressed as aligned dot plot and mean ± SEM. (**B**) Body weight gain curves of AAP-treated mice compared with Veh-AAP. Statistical analysis was performed between vehicle (n = 14) and treated (n = 16) data using one -way ANOVA with repeated measures, followed by Sidak's multiple comparison test. Data are expressed as aligned dot plot and mean ± SEM. ENZ, enzalutamide; AAP, abiraterone acetate-prednisone.

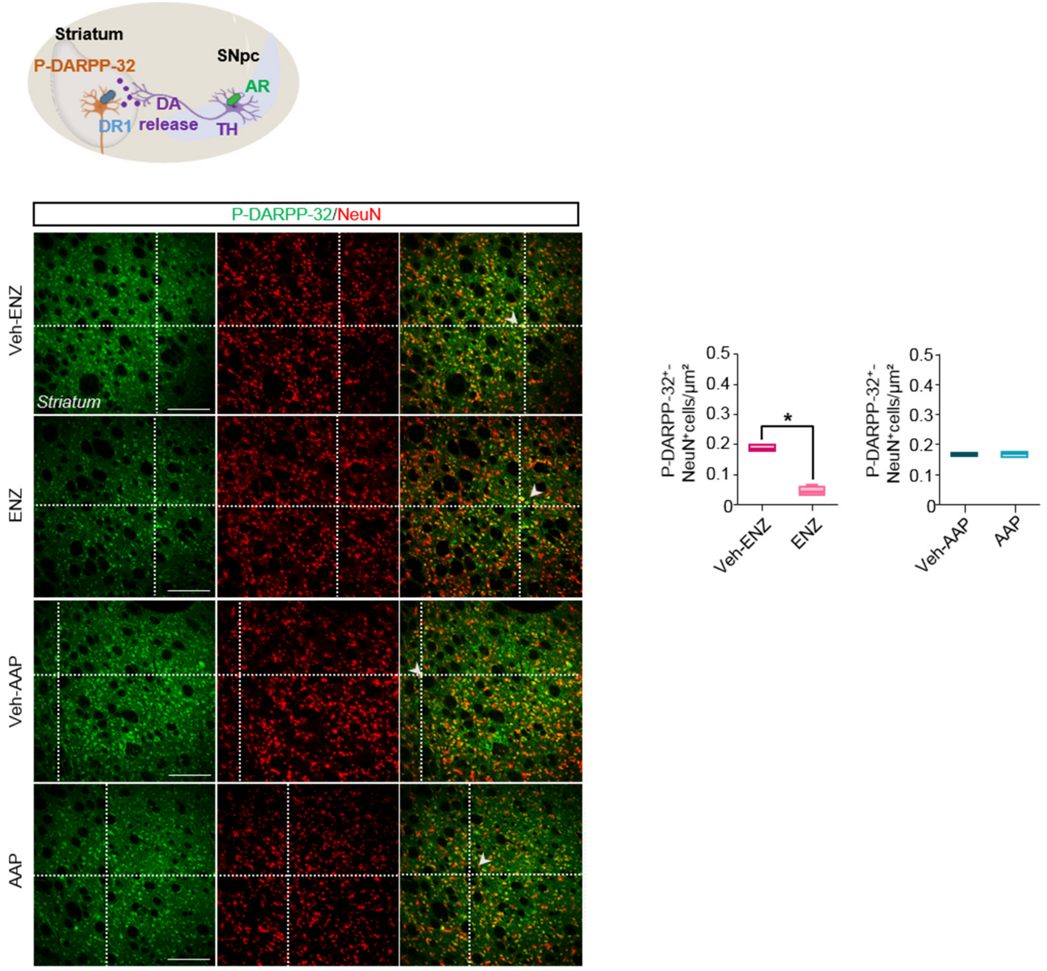

Figure S2. ENZ but not AAP decreases dopaminergic activation of striatal medium spiny neurons. Note. Schematic of SNpC dopaminergic projections to striatum and consequent striatal neuronal activation. Below, representative images and quantification of cAMP-Regulated Neuronal Phosphoprotein (P-DARPP-32, green) and Neuronal Nuclei Antigen (NeuN, red) immunoreactivities in brain striatum of ENZ-, AAP- and Vehicle-treated mice. Box and Whiskers (right panel) represent the number of P-DARPP-32+ / NeuN+ cells in the striatal area of ENZ- and AAP-treated mice compared with respective vehicles. Statistical quantification was performed by using Mann-Whitney test. Data are represented as box and whiskers and mean ± SEM (n = 4 mice), *p < 0.05. Scale bar: 250 µm. ENZ, enzalutamide; AAP, abiraterone acetate-prednisone; P-DARPP-32, cAMP-Regulated Neuronal Phosphoprotein; NeuN, Neuronal Nuclei Antigen.

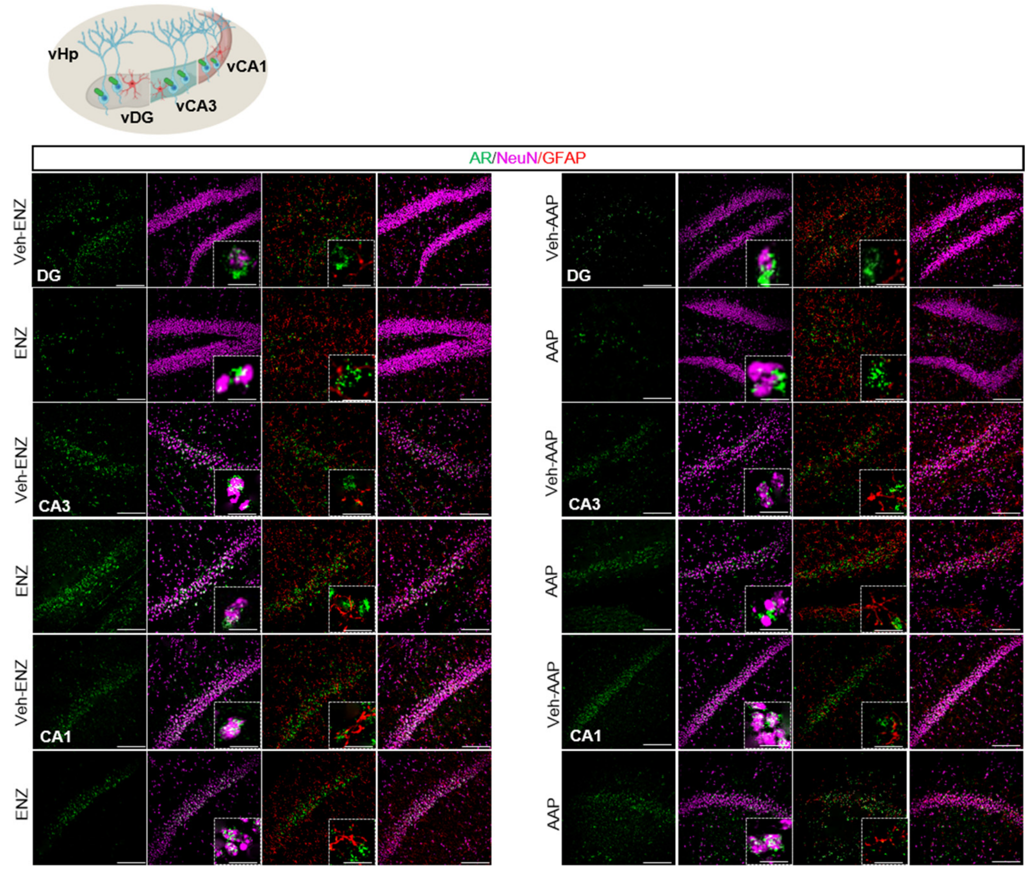

Figure S3. Androgen receptors are expressed by mature neurons of ventral hippocampus. Note. Schematic (**upper panel**) and representative images (**lower panel**) of androgen receptors (AR, green), Neuronal Nuclei Antigen (NeuN, purple) and Glial Fibrillary Acidic Protein (GFAP, red) immunoreactivities in ventral DG (vDG), CA3 (vCA3) and CA1 (vCA1) of ENZ-, AAP- and Vehicle-treated mice. The boxed areas show a magnification of NeuN + /AR+ and the lack of GFAP+ /AR+ cells. Scale bar: 100 µm and 50 µm. AR, androgen receptor; NeuN, Neuronal Nuclei Antigen; GFAP, Glial Fibrillary Acidic Protein; vDG, ventral dentate gyrus; CA3, Cornu ammonis 3; CA1, Cornu ammonis 1; ENZ, enzalutamide; AAP, abiraterone acetate-prednisone.

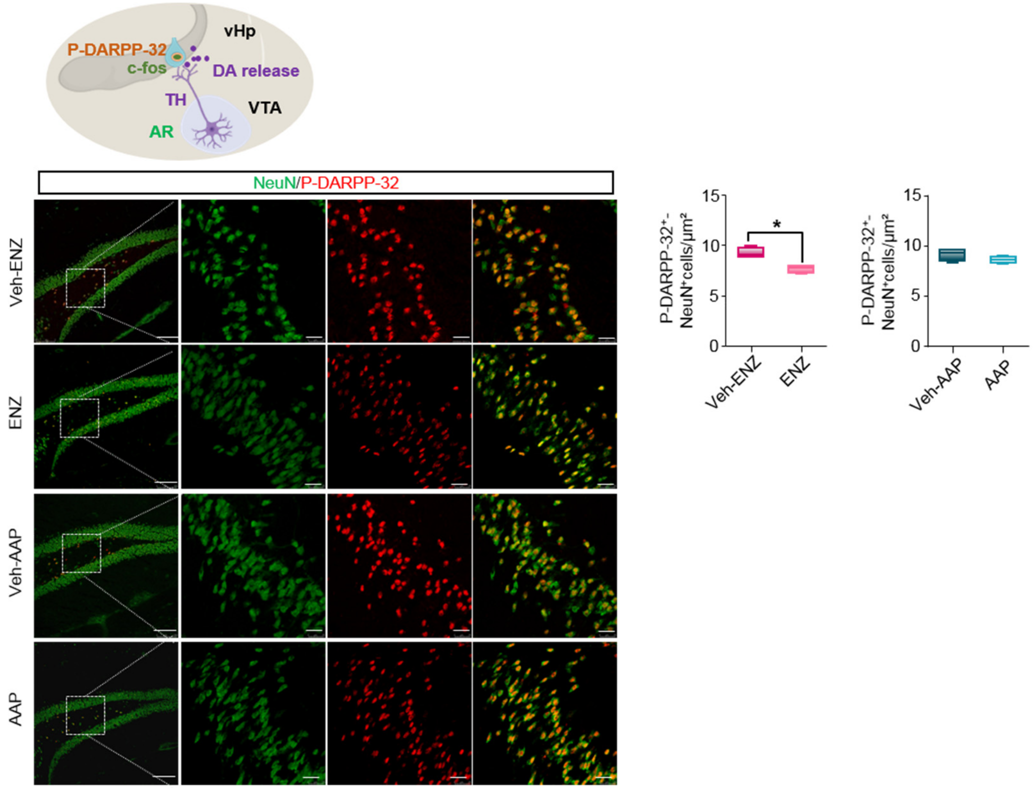

Figure S4. ENZ but not AAP decreases dopaminergic activation of mature neurons of ventral hippocampus. Note. Schematic representation of VTA dopaminergic projections to vHP and consequent neuronal activation. Below, representative images and quantification of NeuN (green) and P-DARPP-32 (red) immunoreactivities in the vDG of ENZ-, AAP- and vehicle-treated mice. A magnification of the squared area shows P-DARPP-32+ /NeuN+ neurons of pyramidal tract of vHp. Box and Whiskers (right panel) represent the number P-DARPP-32+ / NeuN+ cells in the vDG of ENZ- and AAP-treated mice when compared with respective vehicles. Statistical quantification was performed by using Mann-Whitney test. Data are represented as box and whiskers and mean ± SEM ($n = 4$ mice), $*p < 0.05$. Scale bar: 100 µm.and 25 µm. ENZ, enzalutamide; AAP, abiraterone acetate-prednisone; VTA, ventral tegmental area; vHP, ventral hippocampus; NeuN, Neuronal Nuclei Antigen; P-DARPP-32, Phosphorylated form of Dopamine cAMP-Regulated Neuronal Phosphoprotein; vDG, ventral dentate gyrus.

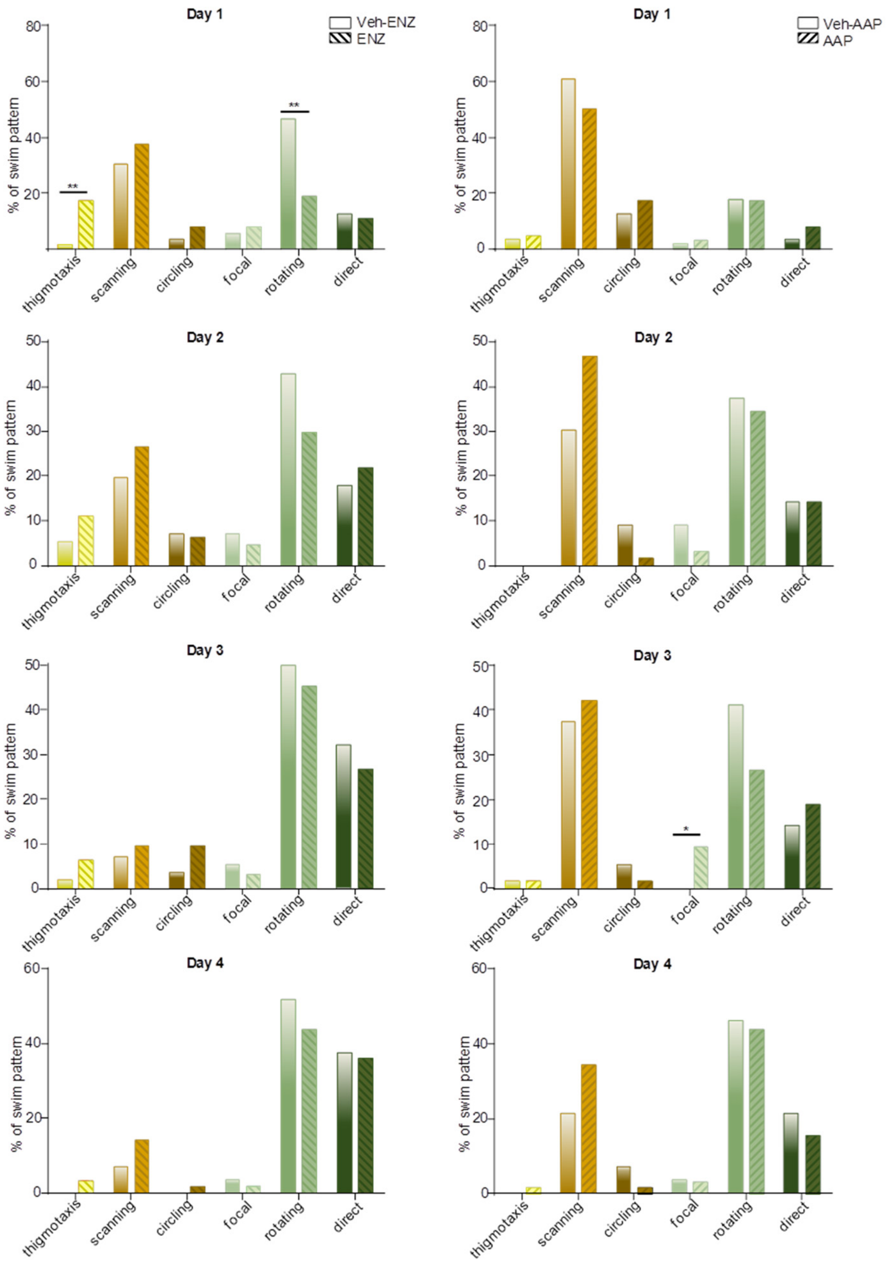

Figure S5. ENZ or AAP treatment impacts on learning strategy trend overtime. Note. Distribution of search-strategies during the 4 days of learning. Statistical comparison of each swim strategy percentage between vehicle (n = 14) and treated (n = 16) mice by Chi-square test with Yates' continuity correction was assessed, each day of learning, from D1 to D4 of ENZ- and AAP-treated mice compared with respective vehicles. Swim paths are presented as histogram plot and percentage, $*p < 0.05$, $**p \leq 0.01$. ENZ, enzalutamide; AAP, abiraterone acetate-prednisone.

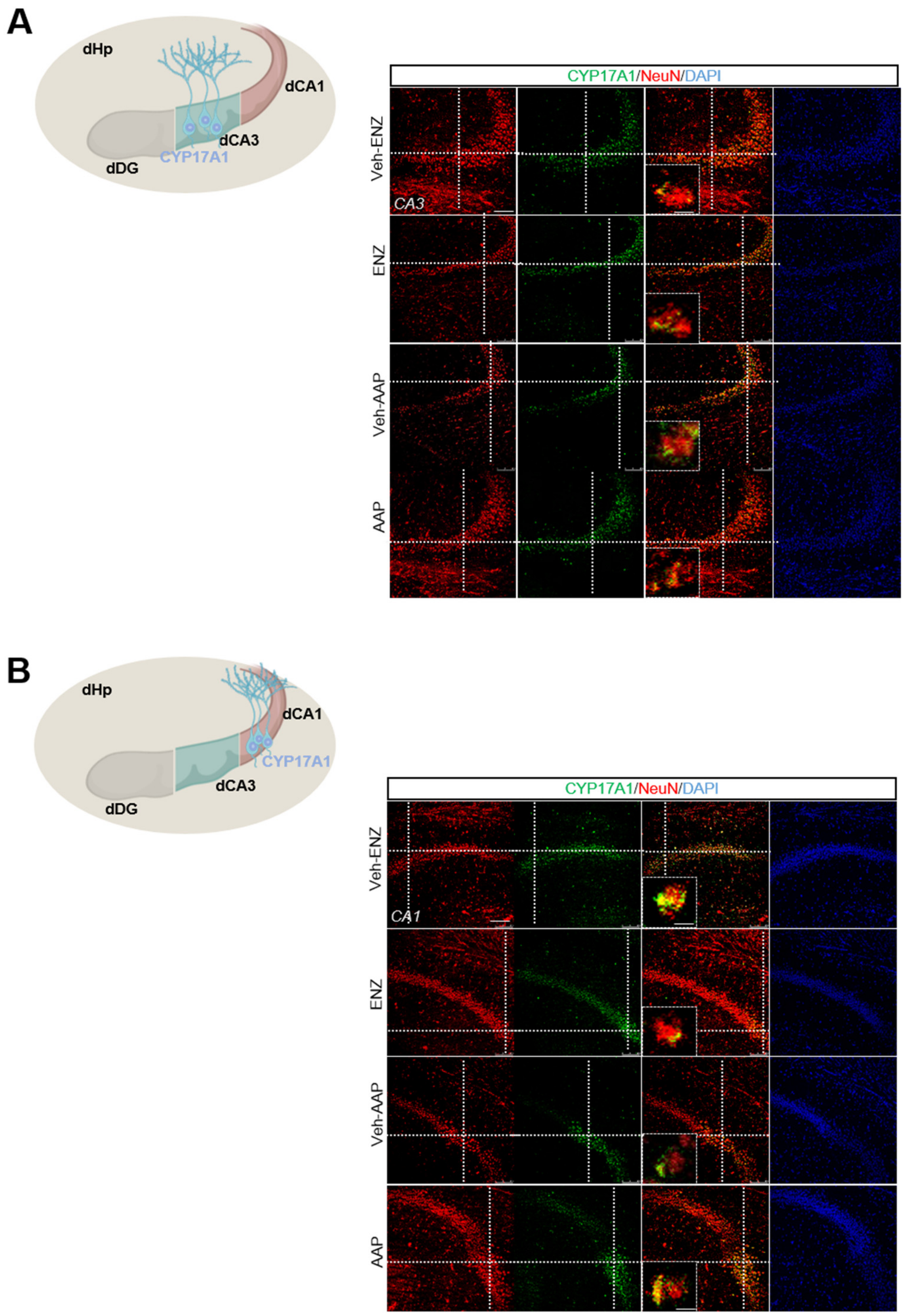

Figure S6. CYP17A1 expression in CA3 and CA1 of dorsal hippocampus. Note. (**A**) Schematic representation of CYP17A1 expression in mature neurons of dCA3. Below, representative example of CYP17A1 (green), NeuN (red) and DAPI (blue) immunoreactivities in the dCA3 of dHP of ENZ-, AAP- and vehicle-treated mice. Intersection of horizontal and vertical lines indicates CYP17A1 + /NeuN+ cells in the 4 conditions of treatment. The boxed areas show a magnification of CYP17A1 + /NeuN+ cells. Scale bar: 100 µm and 50 µm. (**B**) Schematic representation of CYP17A1 expression in mature neurons of dCA1. Below, representative example of CYP17A1 (green), NeuN (red) and DAPI (blue) immunoreactivities in the dCA1 of dHP of ENZ-, AAP- and vehicle-treated mice. Intersection of horizontal and vertical lines indicates CYP17A1 + /NeuN+ cells in the 4 conditions of treatment. The boxed areas show a magnification of CYP17A1 + /NeuN+ cells. Scale bar: 100 µm and 50 µm . CYP17A1, Cytochrome P450 Family 17 Subfamily A Member 1; dCA3, dorsal Cornu Ammonis 3;

NeuN, Neuronal Nuclei Antigen; DAPI, 4′,6-diamidino-2-phenylindol; ENZ, enzalutamide; AAP, abiraterone acetate-prednisone; HP, hippocampus; CA1, cornu ammonis 1.

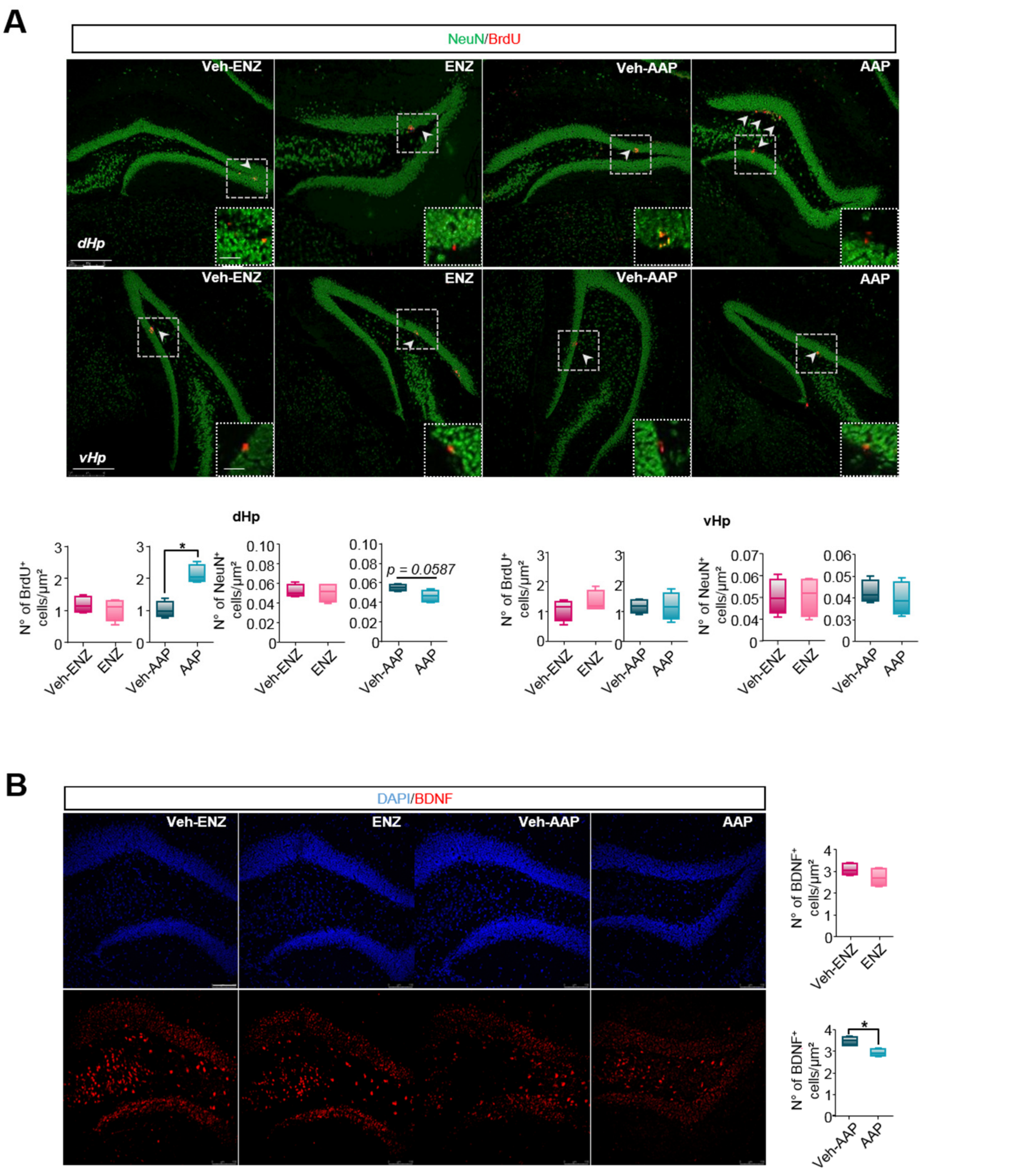

Figure S7. AAP treatment alters neurogenesis with no major effect on mature neurons. Note. (**A**) Representative images and quantification of NeuN (green) and BrdU (red) immunoreactivities in the dorsal (upper panel) and ventral (lower panel) DG of ENZ-, AAP- and vehicle-treated mice. White arrows and the boxed areas show BrdU + -cells and a magnification, respectively. Box and whiskers represent the number of BrdU+ and NeuN+ cells in dorsal (left panel) and ventral (right panel) DG of ENZ and AAP treated mice when compared with respective vehicles. Statistical quantification was performed using Mann-Whitney test. Bars are mean ± SEM (n = 4 mice), *p < 0.05. Scale bar: 100 µm. (**B**) Representative immunolabeling of BDNF (red) (cell nuclei stained with DAPI, blue) and quantification in the dDG of ENZ, AAP and vehicle-treated mice. Statistical quantification was performed using Mann-Whitney test. Bars are mean ± SEM (n = 4 mice), *p < 0.05. Scale bars: 100 µm. AAP, abiraterone acetate-prednisone; NeuN, Neuronal Nuclei Antigen; BrdU, Bromodeoxyuridine; dDG, dorsal dentate gyrus; ENZ, enzalutamide; BDNF, Brain-derived neurotrophic factor; DAPI, 4′,6-diamidino-2-phenylindol.

Table S1. Lack of effect of ENZ or AAP on plasma pro-inflammatory, pluripotent, anti-inflammatory, leukocyte growth and chemotactic cytokines.

Proinflammatory Cytokines	Veh-ENZ	ENZ	Veh-AAPI	AAP	Control	*p* Value
IL-1α	18.9 ± 2.9	23.9 ± 7.7	23.1 ± 6.2	19.7 ± 3.5	13.9 ± 2.7	ns.
IL-1β	19.9 ± 10.2	9.1 ± 1.97	18.1 ± 5.7	7.5 ± 0.78	14.2 ± 4.7	ns.
TNFα	3.18 ± 2.4	0.9 ± 0.3	3.4 ± 1.8	0.4 ± 0.4	0.6 ± 0.4	ns.
IL-12p70	13.1 ± 2.8	10.7 ±1.9	19 ± 6.3	16.9 ± 5.2	11.8 ± 3.1	ns.
Pluripotent cytokines						
IL-2	13.2 ± 1.9	18.9 ± 5.1	22.1 ± 7.8	26.2± 13.3	15.9 ± 3.7	ns.
IL-4	8.6 ± 1.3	6.99 ± 1.9	11.4 ± 3.8	8.4 ± 1.9	8.6 ± 2.8	ns.
IL-6	8.5 ± 1.1	23.4 ± 10.8	11.2 ± 3.7	8.9 ± 2.6	11.5 ± 3.3	ns.
IL-17	8.8 ± 3.5	10.5 ± 4.7	14.7 ±6.7	0.4 ± 13.1	12.3 ± 3.5	ns.
Chemotactic cytokines						
RANTES	15.9 ± 1.6	19.3 ± 2.1	16.9 ± 5.1	12.8 ± 3.2	14.3 ± 2.5	ns.
MIP-1α	2.7 ± 0.0	2.9 ± 0.3	3.1 ± 0.4	2.7 ± 0.0	5.9 ± 2.7	ns.
MCP-1	51.6 ± 9.9	74.0 ± 11.2	59.2 ±12.3	40.8 ± 21.9	39.7 ± 11.7	ns.
Anti-inflammatory cytokines						
IL-3	5.5 ± 1.5	5.4 ± 3.8	4.9 ± 2.1	9.5 ± 5.8	5.2 ± 3.5	ns.
Leukocyte growth cytokines						
IL-10	9.8 ± 2.8	6.1 ± 1.9	4.7 ±2.4	13.1 ± 5.6	2.9 ± 1.0	ns.
GM-CSF	10.5 ± 2.0	10.9 ± 2.9	14.2 ±3.2	12.9 ± 5.5	8.1 ± 1.3	ns.

Note. Circulating cytokines were measured from plasma of Veh-ENZ-, ENZ-, Veh-AAP-, AAP-treated aged castrated (n = 8) and aged non treated non castrated control mice (n = 6). All data are expressed in pg/ml. Not detectable values are expressed as the half of the minimal quantity detected by the kit within this experimental sequence. Statistical quantification among the five groups was assessed using Kruskall Wallis test followed by Dunn's multiple comparison test. Data are expressed as mean ± SEM. GM-CSF Granulocyte Macrophage Colony-stimulating factor; IL Interleukine, MCP-1 Monocyte Chemotactic Protein-1; TNF-α Tumor Necrosis Factor alpha; RANTES Regulated on Activation Normal T cell expressed and secreted; MIP-1α : Macrophage Inflammatory Protein 1-Alpha; MCP-1, Monocyte Chemotactic Protein ; GM-CSF, Granulocyte-Macrophage Colony Stimulating Factor.

Table S2. Summary of the behavioral phenotypes of non-treated non-castred aged mice.

Non-treated Non-Castrated Aged Mice — Spontaneous Activity and Exploratory Behaviors	Variable	Mean ± SEM
Open Field Test		
Control	Vertical activity	46.7 ± 2.499
	Total distance	30.0 ± 1.541
	Total time immobile	200.6 ± 8.835
	Immobile episodes	53.67 ± 3.403
	Grooming time	24.9 ± 4.751
	Center entries	48.33 ± 3.412
	Time in center	116.4 ± 20.43
	Distance in center	8.457 ± 0.9453
	Time immobile in center	36.12 ± 11.62
	Periphery entries	49.17 ± 3.544
	Time in periphery	483.6 ± 20.43
	Distance in periphery	21.55 ± 1.237
	Time immobile in periphery	164.5 ± 16.60
**Anxiety-like behaviors**		
Elevated plus maze		
Control	Distance crossed	3.816 ± 0.5591
	Time immobile	215.8 ± 14.97
	Immobile episodes	38.67 ± 2.753

	SAP	4.833 ± 1.249
	Head dips	18.67 ± 3.051
	% of time in open arms	24.00 ± 12.76
	% of distance crossed in open arms	23.17 ± 9.382
Light dark box		
Control	Entries in the light box	7.167 ± 1.887
	Time in the light box	58.25 ± 18.36
	Latency to the first entry in the light box	193.3 ± 65.71
**Depressive-like behaviors**		
Tail suspension test		
Control	Immobility duration	175.1 ± 175.1
	Latency to the first immobile episode	78.82 ± 5.497
Forced swim test		
Control	Immobility duration	163.5 ± 15.05
	Latency to the first immobile episode	169.9 ± 15.28

Note. Different items were analyzed in the open field test, the elevated plus maze test, the light and dark box test, the tail suspension test, the forced swim test in aged non castred non treated control mice (n = 6). Data are expressed as mean ± SEM.

Table S3. Summary of the behavioral phenotypes of aged castred mice treated with ENZ or AAP.

**NGT-Treated Aged Castrated Mice** / **Spontaneous Activity and Exploratory Behaviors**	**Variable**	**Mean ± SEM**	**T_{DFn} or $F_{DFn,DFd}$ or U_{DFn}**	***p* Value**
Open field test				
Veh-AAP vs. AAP	Vertical activity	34.86 ± 3.477 vs. 29.94 ± 2.586	t_{28}= 1.153	p = 0.2586
	Total distance	22.23 ± 1.367 vs. 20.71 ± 1.367	t_{28}= 0.7839	p = 0.4397
	Total time immobile	269.9 ± 15.67 vs. 306.5 ±17.26	t_{28}= 1.554	p = 0.1314
	Immobile episodes	60.79 ± 2.557 vs. 59.81 ± 2.370	t_{28}= 0.2793	p = 0.7821
	Grooming time	19.49 ± 2.132 vs. 12.73 ± 1.229	t_{28}= 2.835	**p = 0.0084**
	Center entries	41.50 ± 3.278 vs. 36.19 ± 3.168	t_{28}= 1.163	p = 0.2547
	Time in center	133.5 ± 10.30 vs. 135.5 ± 14.44	U_{28}= 104.5	p = 0.7673
	Distance in center	7.644 ± 0.6105 vs. 6.658 ± 0.6097	t_{28}= 1.138	p = 0.2646
	Time immobile in center	54.19 ± 9.125 vs. 68.16 ± 11.57	U_{28}= 92	p = 0.4232
	Periphery entries	41.29 ± 3.304 vs. 36.31 ± 3.162	t_{28}= 1.086	p = 0.2868
	Time in periphery	466.5 ± 10.30 vs. 464.5 ± 14.44	U_{28}= 104.5	p = 0.7673
	Distance in periphery	14.59 ± 0.9904 vs. 14.05 ± 0.9962	t_{28}= 0.3729	p = 0.7073
	Time immobile in periphery	215.6 ± 12.78 vs. 244.6 ± 18.29	t_{28}= 1.261	p = 0.2176
Veh-ENZ vs. ENZ	Vertical activity	49.62 ± 4.3999 vs. 34.67 ± 2.433	t_{28}= 3.081	**p = 0.0048**
	Total distance	19.54 ± 1.212 vs. 15.48 ± 0.8989	t_{28}= 2.037	**p = 0.0468**

	Total time immobile	251.9 ± 15.68 vs. 317.3 ± 14.92	t_{28}= 3.016	***p* = 0.0057**
	Immobile episodes	56.54 ± 2.667 vs. 57.33 ± 2.341	t_{28}= 0.2250	*p* = 0.8238
	Grooming time	26.68 ± 3.771 vs. 29.51 ± 3.913	t_{28}= 0.5152	*p* = 0.6108
	Center entries	26.69 ± 1.834 vs. 18.67 ± 2.042	t_{28}= 2.2886	***p* = 0.0077**
	Time in center	49.41 ± 5.668 vs. 36.72 ± 4.777	t_{28}= 1.724	*p* = 0.0965
	Distance in center	3.224 ± 0.2445 vs. 2.729 ± 0.3111	t_{28}= 0.9668	*p* = 0.3419
	Time immobile in center	13.11 ± 4.368 vs. 8.525 ± 2.105	U_{28}= 95.50	*p* = 0.5403
	Periphery entries	26.92 ± 1.893 vs. 18.44 ± 1.962	t_{28}= 3.067	***p* = 0.0049**
	Time in periphery	550.6 ± 5.669 vs. 563.9 ± 4.514	t_{28}= 1.368	*p* = 0.0731
	Distance in periphery	16.32 ± 1.018 vs. 12.47 ± 0.7010	t_{28}= 3.202	***p* = 0.0035**
	Time immobile in periphery	237.8 ± 15.69 vs. 313 ± 15.10	t_{28}= 3.430	***p* = 0.0093**
**Anxiety-like behaviors**				
Elevated plus maze				
Veh-AAP vs. AAP	Distance crossed	4.376 ± 0.3012 vs. 4.870 ± 0.3170	t_{28}= 1.12	*p* = 0.2721
	Time immobile	211.1 ± 6.169 vs. 207 ± 6.406	t_{28}= 0.4596	*p* = 0.6949
	Immobile episodes	35 ± 1.671 vs. 39 ± 1.886	t_{28}= 1.567	*p* = 0.1283
	SAP	3.5 ± 0.9361 vs. 3.125 ± 0.7004	t_{28}= 0.3256	*p* = 0.7471
	Head dips	12.64 ± 1.830 vs. 17.38 ± 1.248	t_{28}= 2.182	***p* = 0.0376**
	% of time in open arms	17.14 ± 5.130 vs. 22.63 ± 4.904	U_{28}= 89.50	*p* = 0.3588
	% of distance crossed in open arms	21.79 ± 4.497 vs. 21.31 ± 3.931	U_{28}= 107.5	*p* = 0.8616
Veh-ENZ vs. ENZ	Distance crossed	4.347 ± 0.4035 vs. 3.818 ± 0.3838	t_{28}= 0.9957	*p* = 0.3279
	Time immobile	206.8 ± 8.456 vs. 218.2 ± 7.562	t_{28}= 0.901	*p* = 0.3753
	Immobile episodes	38.07 ± 2.822 vs. 37.29 ± 2.230	t_{28}= 0.5042	*p* = 0.6181
	SAP	14 ± 1.383 vs. 13.44 ± 1.505	t_{28}= 0.2723	*p* = 0.7874
	Head dips	14.43 ± 1.806 vs. 11.25 ± 1.442	t_{28}= 0.2723	*p* = 0.7874
	% of time in Open Arms	24.22 ± 3.555 vs. 20.15 ± 4.310	U_{28}= 85	*p* = 0.2751
	% of distance crossed in open arms	25.63 ± 3.176 vs. 18.38 ± 3.015	t_{28}= 1653	*p* = 0.1095
Light/dark box				
Veh-AAP vs. AAP	Entries in the light box	4.429 ± 0.7010 vs. 5.313 ± 0.6565	t_{28}= 0.9203	*p* = 0.3653
	Time in the light box	26.26 ± 4.74 vs.	t_{28}= 1.625	*p* = 0.1153

	Latency to the first entry in the light box	38.78 ± 5.884<br>213.3 ± 37.67 vs. 158.7 ± 25.85	t_{28}= 1.209	$p = 0.1794$
Veh-ENZ vs. ENZ	Entries in the light box	8.286 ± 1.404 vs. 6.188 ± 0.7595	t_{28}= 1.360	$p = 0.4140$
	Time in the light box	57.34 ± 9.305 vs. 50.44 ± 8.887	t_{28}= 0.5367	$p = 0.1560$
	Latency to the first entry in the light box	139.6 ± 22.76 vs. 142.3 ± 32.87	t_{28}= 0.00639	$p = 0.9476$
**Depressive-like behaviors**				
Tail suspension test				
Veh-AAP vs. AAP	Immobility duration	169.9 ± 11.31 vs. 163.2 ± 8.347	t_{28}= 0.568	$p = 0.5746$
	Latency to the first immobile episode	103.8 ± 9.167 vs. 90.13 ± 8.161	t_{28}= 1.268	$p = 0.2125$
Veh-ENZ vs. ENZ	Immobility duration	141.9 ± 7.083 vs. 161.3 ± 8.677	t_{28}= 2.357	$\mathbf{p = 0.0256}$
	Latency to the first immobile episode	73.85 ± 6.016 vs. 73.53 ± 6.938	t_{28}= 0.2267	$p = 0.8223$
Forced swim test				
Veh-AAP vs. AAP	Immobility duration	201.2 ± 19.60 vs. 190.3 ± 18.76	t_{28}= 0.3993	$p = 0.6670$
	Latency to the first immobile episode	117.2 ± 12.94 vs. 128.7 ± 12.53	t_{28}= 0.5263	$p = 0.6028$
Veh-ENZ vs. ENZ	Immobility duration	206.3 ± 12.74 vs. 196.8 ± 14.26	t_{28}= 0.857	$p = 0.3987$
	Latency to the first immobile episode	129.6 ± 15.88 vs. 136.9 ± 15.69	t_{28}= 0.1279	$p = 0.8991$
**Spatial learning and memory**				
Morris water maze				
Veh-AAP vs. AAP	Escape latency (familiarization)	32.99 ± 3.315 vs. 35.23 ± 2.926	t_{28}= 0.5087	$p = 0.6150$
	Distance crossed (familiarization)	5.417 ± 0.6168 vs. 6.067 ± 0.6149	U_{28}= 97	$p = 0.5521$
	Mean speed (familiarization)	0.1654 ± 0.006 vs. 0.1720 ± 0.005	U_{28}= 97.50	$p = 0.5589$
	Escape latency (learning)			
	Day 1	40.839 ± 3.226 vs. 40.344 ± 3.119	Trt:$F_{1,28}$ = 1.131	$p = 0.2966$
	Day 2	24.586 ± 3.275 vs. 28.405 ± 3.484	Day:$F_{3,84}$ = 19.16	$p < 0.0001$
	Day 3	23.345 ± 2.282 vs. 27.612 ± 3.074		
	Day 4	18.029 ± 2.689 vs. 21.063 ± 2.891	Int:$F_{3,84}$ = 0.2816	$p = 0.8386$
	Distance crossed (learning)			
	Day 1	7.621 ± 0.565 vs. 7.545 ± 0.662	Trt:$F_{1,28}$ = 0.9956	$p = 0.3269$
	Day 2	4.284 ± 0.519 vs. 5.397 ± 0.678	Day:$F_{3,84}$ = 21.38	$p < 0.0001$
	Day 3	4.526 ± 0.445 vs. 5.048 ± 0.532		
	Day 4	3.417 ± 0.504 vs. 3.857 ± 0.527	Int:$F_{3,84}$ = 0.4517	$p = 0.7167$

	Mean speed (learning)			
	Day 1	0.207 ± 0.014 vs. 0.207 ± 0.013	Trt:$F_{1,28}$ = 1.490	p = 0.2324
	Day 2	0.214 ± 0.029 vs. 0.197 ± 0.007		
	Day 3	0.240 ± 0.020 vs. 0.211 ± 0.015	Day:$F_{3,84}$ = 0.6293	p = 0.5981
	Day 4	0.208 ± 0.015 vs. 0.0208 ± 0.012	Int:$F_{3,84}$ = 0.3313	p = 0.8027
	Duration (probe test)	35.93 ± 2.567 vs. 31.75 ± 1.377	Trt:$F_{1,28}$ = 1.486	p = 0.1485
	Distance (probe test)	33.21 ± 2.427 vs. 30.19 ± 1.330	Trt:$F_{1,28}$ = 1.131	p = 0.2676
	% of time spent in NW quadrant (retrieval test)	21.19 ± 3.180 vs. 23.48 ± 2.143	t_{28}= 0.6118	p = 0.5456
	% of distance crossed in the NW quadrant (retrieval test)	3.901 ± 0.5951 vs. 4.191 ± 0.3489	t_{28}= 0.4329	p = 0.6684
	Escape latency (flexibility)	46.95 ± 4.715 vs. 45.11 ± 4.398	U_{28}= 99	p = 0.5753
	Distance crossed (flexibility)	8.792 ± 0.7359 vs. 9.650 ± 0.9469	t_{28}= 0.7006	p = 0.4894
	Mean speed (flexibility)	0.2029 ± 0.01436 vs. 0.1943 ± 0.008442	U_{28}= 105.5	p = 0.7974
	Escape latency (familiarization)	48.21 ± 3.233 vs. 47.76 ± 1.871	t_{28}= 0.1242	p = 0.9020
	Distance crossed (familiarization)	8.263 ± 0.7647 vs. 8.279 ± 0.4464	t_{28}= 0.0180	p = =0.9857
	Mean speed (familiarization)	0.1642 ± 0.009 vs. 0.1714 ± 0.006	t_{28}= 0.6618	p = 0.5135
	Escape latency (learning)		Trt:$F_{1,28}$ = 2.596	p = 0.1183
	Day 1	37.655 ± 2.775 vs. 43.853 ± 3.462	t_{28}= 0.6618	p = 0.5135
	Day 2	30.862 ± 4.004 vs. 34.938 ± 3.251		
	Day 3	19.098 ± 3.203 vs. 27.073 ± 3.351	Day:$F_{3,84}$ = 35.16	p < 0.0001
	Day 4	15.485 ± 1.924 vs. 17.847 ± 2.612	Int:$F_{3,84}$ = 0.4674	p = 0. 7058
	Distance crossed (learning)			
Veh-ENZ vs. ENZ	Day 1	6.581 ± 0.527 vs. 7.242 ± 0.589	Trt:$F_{1,28}$ = 2.131	p = 0.1555
	Day 2	5.185 ± 0.692 vs. 5.804 ± 0.549		
	Day 3	3.185 ± 0.460 vs. 4.559 ± 0.612	Day:$F_{3,84}$ = 27.65	p < 0.0001
	Day 4	2.772 ± 0.394 vs. 3.157 ± 0.474	Int:$F_{3,84}$ = 0.4124	p = 0.7445
	Mean speed (learning)			
	Day 1	0.176 ± 0.008 vs. 0.168 ± 0.007	Trt:$F_{1,28}$ = 0.1416	p = 0.7095
	Day 2	0.172 ± 0.006 vs. 0.166 ± 0.010	Day:$F_{3,84}$ = 51.72	p = 0.6715
	Day 3	0.172 ± 0.010 vs.		

Day 4	0.172 ± 0.009<br>0.176 ± 0.008 vs.<br>0.176 ± 0.007	Int:$F_{3,84}$ = 0.3018	p = 0.8240
% of time spent in NW quadrant (probe test)	40.89 ± 2.830 vs.<br>39.82 ± 2.757	U_{28}= 109	p = 0.9185
% of distance crossed in the NW quadrant (probe test)	39.79 ± 2.459 vs.<br>38.42 ± 2.496	U_{28}= 110	p = 0.9510
% of time spent in NW quadrant (retrieval test)	13.75 ± 2.531 vs.<br>18.35 ± 2.800	t_{28}= 1.206	p = 0.2378
% of distance crossed in the NW quadrant (retrieval test)	2.532 ± 0.4029 vs.<br>3.598 ± 0.6047	t_{28}= 1.423	p = 0.1657
Escape latency (flexibility)	34.50 ± 5.496 vs.<br>29.33 ± 5.819	U_{28} = 97	p = 0.5409
Distance crossed (flexibility)	6.936 ± 1.088 vs.<br>4.935 ± 1.053	t_{28}= 1.318	p = 0.1983
Mean speed (flexibility)	0.2032 ± 0.009 vs.<br>0.1797 ± 0.012	t_{28}= 1.473	p = 0.1519

Note. Different items were analyzed in the open field test, the elevated plus maze test, the light and dark box test, the tail suspension test, the forced swim test and the Morris water maze in mice treated with veh-AAP (n = 14), AAP (n = 16), veh-ENZ (n = 14), ENZ (n = 16). Based on the group normality, data were analyzed using either: (i) one way ANOVA with repeated measures and t-test for parametric analysis or Mann-Whitney test for non parametric analysis. Behavioral data are expressed as mean ± SEM and statistical data are indicated as t or F value in case of parametric tests, while U value is indicated in case of non parametric test. In ANOVA analysis, the presented factors are treatment (Trt) and days and their interacion (Int). $p < 0.05$ was considered as significant.
